# “A Baby Was an Added Burden”: Predictors and Consequences of Unintended Pregnancies for Female Sex Workers in Mombasa, Kenya: A Mixed-Methods Study

**DOI:** 10.1371/journal.pone.0162871

**Published:** 2016-09-30

**Authors:** Stanley Luchters, Wilkister Bosire, Amy Feng, Marlise L. Richter, Nzioki King’ola, Frances Ampt, Marleen Temmerman, Matthew F. Chersich

**Affiliations:** 1 Burnet Institute, Melbourne, Australia; 2 International Centre for Reproductive Health (ICRH), Department of Obstetrics and Gynaecology, Ghent University, Ghent, Belgium; 3 Department of Epidemiology and Preventive Medicine, School of Public Health and Preventive Medicine, Faculty of Medicine, Nursing and Health Sciences, Monash University, Melbourne, Australia; 4 International Centre for Reproductive Health (ICRH), Mombasa, Kenya; 5 Department of Obstetrics and Gynaecology, The Royal Women’s Hospital, Melbourne, Australia; 6 Department of Public Health and Family Medicine, University of Cape Town, Cape Town, South Africa; 7 African Centre for Migration & Society, University of the Witwatersrand, Johannesburg, South Africa; 8 Department of Reproductive Health Research, World Health Organization, Geneva, Switzerland; 9 Centre for Health Policy, School of Public Health, Faculty of Health Sciences, University of the Witwatersrand, Johannesburg, South Africa; 10 Wits Reproductive Health and HIV Institute, Faculty of Health Sciences, University of the Witwatersrand, Johannesburg, South Africa; Vanderbilt University, UNITED STATES

## Abstract

**Introduction:**

Female sex workers (FSW) have high rates of unintended pregnancy, sexually transmitted infections including HIV, and other adverse sexual and reproductive health outcomes. Few services for FSWs include contraception. This mixed-methods study aimed to determine the rate, predictors and consequences of unintended pregnancy among FSWs in Mombasa, Kenya.

**Methods:**

A prospective cohort study of non-pregnant FSWs was conducted. Quantitative data were collected quarterly, including a structured questionnaire and testing for pregnancy and HIV. Predictors of unintended pregnancy were investigated using multivariate logistic regression. Qualitative data were gathered through focus group discussions and in-depth interviews with FSWs who became pregnant during the study, and interviews with five key informants. These data were transcribed, translated and analysed thematically.

**Results:**

Four hundred women were enrolled, with 92% remaining in the cohort after one year. Fifty-seven percent reported using a modern contraceptive method (including condoms when used consistently). Over one-third (36%) of women were using condoms inconsistently without another method. Twenty-four percent had an unintended pregnancy during the study. Younger age, having an emotional partner and using traditional or no contraception, or condoms only, were independent predictors of unintended pregnancy. Women attributed pregnancy to forgetting to use contraception and being pressured not to by clients and emotional partners, as well as “bad luck”. They described numerous negative consequences of unintended pregnancy.

**Conclusion:**

Modern contraceptive uptake is surprisingly low in this at-risk population, which in turn has a high rate of unintended pregnancy. The latter may result in financial hardship, social stigma, risk of abandonment, or dangerous abortion practices. FSWs face considerable barriers to the adoption of dual method contraceptive use, including low levels of control in their emotional and commercial relationships. Reproductive health services need to be incorporated into programs for sexually transmitted infections and HIV, which address the socially-determined barriers to contraceptive use.

## Introduction

Nearly one in twenty women in sub-Saharan Africa exchange sex for money, goods or favours[1], with similar rates observed in Mombasa and throughout Kenya[2, 3]. Sex work remains criminalised across most of Africa, including in Kenya, and is a highly stigmatised practice that exacerbates the vulnerability already experienced by women in many settings. Stigma and criminalisation limit female sex workers’ (FSWs) access to health services, including for reproductive health, and for testing and treatment of sexually transmitted infections (STIs) and HIV[4, 5]. This contributes to disproportionately poor sexual and reproductive health (SRH) outcomes in this population.

Estimates of contraceptive use vary widely between studies, and are complicated by the different indicators collected. A systematic review by Scorgie et al[4] found that consistent condom use by Kenyan FSWs ranged from 15% to 38% with emotional partners and from 29% to 74% with clients. Use of contraceptives other than condoms is measured less frequently in studies of FSWs, but includes estimates of 50% in Uganda[6], 16% in Madagascar[7], and 40% in Mombasa.

The available evidence indicates that more than two-thirds of FSWs in sub-Saharan Africa have at least one child, with estimates in Kenya closer to 90%[4, 8, 9]. FSWs in the region also experience high rates of unintended pregnancy, with 12-month cumulative incidence rates of around 25%[7, 10], and a 52% lifetime rate reported in Mombasa[11]. As many FSWs come from vulnerable circumstances with limited economic options, unintended pregnancy further perpetuates the cycle of stigma and socio-economic vulnerability, and consequently may deepen their dependency on sex work[8, 12].

Among the general population of (married) women in Kenya, modern contraceptive use has increased in recent years, from 39% in 2009 to 53% in 2014[13]. There has been increasing commitment to family planning at the national level, with the right to reproductive health enshrined in the new constitution in 2010. However, decentralization to county governments is claimed to have fragmented the funding and provision of contraceptive services[14]. Furthermore, it is not known whether the gains in family planning coverage have translated into improved uptake among FSWs and other high risk groups.

While FSWs are frequently targeted by HIV and other STI prevention programs[8, 15, 16], such programs often overlook their broader reproductive health needs and the context in which sex work takes place[17, 18]. A review of SRH projects among FSWs in Africa found that while condom provision was almost universal, only 7 of 54 projects included other contraceptive services, and few specifically promoted dual method use (the concurrent use of condoms and another effective contraceptive method). Six programs offered pregnancy testing and none reported providing pregnancy termination services[19]. The lack of synergy between HIV prevention and family planning services also has significant implications for mother-to-child transmission of HIV[18, 20, 21]. Preventing unintended pregnancy among the estimated 35% of FSWs living with HIV, many of whom do not have access to abortion, will not only reduce personal hardship, but is also a cost-effective strategy to reduce paediatric HIV[8, 22].

Termination of pregnancy is restricted in Kenya and is only legal if the pregnancy is thought to endanger the woman’s health. Access to safe termination of pregnancy is severely limited, and rates of morbidity and mortality are high. It is estimated that over 450,000 women in Kenya had an induced abortion in 2012, and of these, 120,000 were treated for complications. The case fatality rate is approximately 266 per 100,000 unsafe abortions[23].

Women who proceed with an unintended pregnancy face significant health risks. Sub-Saharan Africa continues to have the highest maternal mortality ratio (MMR) by region, with 546 maternal deaths per 100,000 live births, compared to just 12 in developed regions[24]. In Kenya, only 58% of women access the recommended four visits of antenatal care, 62% have a skilled birth attendant at delivery[25], and there are 510 maternal deaths per 100,000 live births[24].

This prospective mixed-methods observational study aimed to determine the rate, predictors and consequences of unintended pregnancy among a cohort of FSWs in Mombasa, Kenya. The study also assessed rates and predictors of contraceptive use, as well as perceptions and attitudes towards family planning and abortion.

## Methods

### Study setting and population

Mombasa is the second largest city in Kenya with a population of over one million people. It is an important economic and trade hub, with a large international airport and seaport serving Kenya and neighbouring countries. It also has a long-standing tourist industry, attracting large numbers of visitors each year. The study focussed on two divisions (Kisauni and Changamwe) in Mombasa county, which were purposively selected based on previous experience working in these areas and proximity to the research NGO. The main economic activity in coastal Kisauni revolves around tourism, whilst Changamwe, encompassing the harbour, airport and the start of the Trans East African highway, is an important route for trucks and private vehicles. It is estimated that some 9,300 FSWs live and work in Mombasa[3].

### Data collection and assessments

Our mixed-methods study consisted of a prospective cohort and serial embedded qualitative inquiries. Detailed outline of the methodology of the cohort study is provided elsewhere[26, 27]. In brief, trained field workers were assigned to 11 zones within Kisauni and Changamwe, and were responsible for inviting women to enrol in the study and maintaining contact with them throughout the study. Convenience sampling was used and FSWs were recruited in their homes, guesthouses and streets.

To be eligible, FSWs had to be aged 16 years or older, provide adequate locator information for tracing, be HIV negative based on laboratory testing, and not currently be pregnant as assessed by self-report and confirmed by negative pregnancy screening test. Women aged 16 or 17 years who were eligible for the study were considered to be mature minors and able to consent for participation in the study. Given the sensitive and stigmatising nature of the subject matter, next of kin, caretakers, or guardians were not asked to consent on behalf of 16- and 17-year-old participants. All participants, including those aged less than 18 years, had to be able and willing to provide written informed consent for study participation. Illiterate volunteers could provide a thumbprint witnessed by an independent person of their choosing, who was able to affirm that informed consent had taken place without coercion.

Those planning to travel or relocate from the study areas, or participating in other HIV intervention studies, were excluded from participation. Participants could voluntarily withdraw from the study at any time; site investigators could also withdraw volunteers from the study in order to protect their safety or if they were unable or unwilling to comply with study procedures. External monitoring was conducted, verifying available source documentation with study-specific clinical record forms.

Visits for screening and enrolment were followed by quarterly study visits until 12 months post-enrolment. Study visits took place at a primary health centre in Changamwe and an FSW drop-in centre in Kisauni. Informed consent, collection of baseline characteristics including demographics and STI symptoms, and testing for HIV and pregnancy was performed at screening (visit 1).

A structured questionnaire was administered at each of the five study visits to collect data on socio-demographics (baseline), sexual behaviour, relationship characteristics, pregnancy intention and family planning use. Local staff translated the English questionnaires into Swahili, which were field tested before use. Questionnaires were administered in English or Swahili depending on the preference of the study participant. At least four attempts were made to locate participants who defaulted on follow up.

Pregnancy and HIV testing was done at each quarterly visit. A single positive urine test was considered diagnostic of pregnancy. Contraceptive and risk-reduction counselling were also provided at each visit. Contraceptives, including male and female condoms, were offered free of charge. The study did not include provision of access to abortion services, or specific counselling for women who had an unintended or unwanted pregnancy. Gynaecological examination and sexually transmitted infection (STI) testing were done at baseline and 12 months, as well as when clinically indicated. STI and HIV testing and management procedures have been described elsewhere[26].

Qualitative inquiries aimed to explore FSWs’ attitudes towards and experiences of family planning, unintended pregnancy and abortion. Four focus group discussions were held with 6–10 randomly selected cohort participants, separated by age group (16–20, 20–24, 25–29 and 30–34 years, consistent with Kenyan Demographic and Health Survey methodology[28]). FSWs who had become pregnant during the course of the cohort study were purposively sampled by the study team to take part in 20 in-depth interviews. Participants were selected based on whether the research team felt they could contribute interesting perspectives. Participants were invited to partake by female peer educators who were part of the study team, and well known in the local sex work community. focus group discussions and interviews were held at the research clinic and drop-in centres based within the community. A moderator and note taker employed by ICRH and experienced in qualitative research conducted each focus group discussion, prompted by pilot-tested guides. In addition, five key informant interviews were scheduled with one clinician, one nurse, one research assistant and two community field workers. The number of key expert interviews was chosen to allow for a detailed examination of the main themes that emerged during interviews and group discussions. No repeat interviews were done, and transcripts were not shared with the participants.

The Kenyatta National Hospital Ethics and Research Committee reviewed and gave approval for the full study, including for the collection of consent from minors without guardian consent on their behalf (P199/11/2005).

### Study measures

A FSW was defined as a woman reporting to have had sexual intercourse at least once in the past three months, and having received money in exchange for it as part of her livelihood in the last six months. Pregnancy intention was assessed at each visit for each type of sexual partner: emotional partners such as boyfriends and husbands; regular clients; and casual clients. Unintended pregnancy was defined as any positive pregnancy test among women who reported no intention to become pregnant in that period.

Contraceptive use was assessed and described by type. Modern method use is defined as oral contraceptives, implants, injectables, intra-uterine devices (IUD), male and female condoms (self-reported consistent use with all partners over the past 3 months), sterilization, Lactational Amenorrhoea Method, and emergency contraceptives, as per the World Health Organization definition[29]. IUDs and implants were further categorised as long-acting reversible contraceptives (LARCs). Adherence to user-dependent contraceptive methods was not assessed. The term traditional contraceptive methods was used to describe the use of withdrawal or fertility awareness methods such as the use of menstrual cycle, and symptom-based methods such as monitoring cervical mucus and body temperature. Dual method use was defined as the consistent use of condoms concurrently with another modern method of contraception. Having an abortion was defined as having had either a miscarriage or any method of pregnancy termination.

Emotional partner was defined as a person with whom the FSW has a sexual relationship and an emotional attachment and is not dependent on the exchange of money for sex every time, e.g. a boyfriend or husband. FSWs’ subjective experiences of being controlled by an emotional partner was assessed by an adaptation[30] of the Relationship Control Subscale from the Sexual Relationship Power Scale[31]. This consisted of a 12-item questionnaire with a four-point Likert scale for each item, ranging from 1 (strongly agree) to 4 (strongly disagree). Total cumulative scores were categorized as low relationship control (score 12–24), medium control (score 25–36) and high control (37–48)[27].

Alcohol used was assessed using the Alcohol Use Disorders Identification Test (AUDIT) criteria and categorized as abstinence; low-risk drinking (AUDIT score 1–7); hazardous drinking (AUDIT score of 8–15); and harmful drinking or alcohol dependence (AUDIT score of ≥16)[32].

### Data management and analysis

Data were double entered by separate clerks and analysed using Stata SE 12.0 (Stata Corporation, College Station, TX, USA). Descriptive analysis of population characteristics assessed the distribution of continuous variables and the frequency distribution of categorical variables in contingency tables, stratified by current use of modern contraceptives. Associations were assessed between current modern contraceptive use, and demographic, sex work and reproductive health characteristics at cohort entry. Chi-square tests were used for analysis of categorical variables, while for continuous variables we used Student’s *t* test (variables with a Normal distribution) or Wilcoxon rank sum tests (non-Normal variables). Using multivariable logistic regression, we investigated whether socio-demographic or reproductive predictors were independently associated with the period prevalence of unintended pregnancy during the 12-month follow-up.

Data were collected from in-depth interviews and focus group discussions via note-taking and audio-recording. Tape recordings were transcribed and translated verbatim. Data were typed in plain text and imported into Nvivo 7 for thematic analysis. One experienced female qualitative researcher coded the data, drawing initial categories (nodes) and emerging themes from the transcripts and notes. Pseudonyms were assigned to each participant in the qualitative study, and are reported in this manuscript, preserving participants’ anonymity. Interviews were analysed thematically, following a grounded-theory approach. This process involved initial open coding of transcripts, then identification of key concepts that best summarised and explained the participants’ experiences. Key concepts were then used to guide the remainder of the analysis and writing up of the data. The conceptual framework was cross-checked by other members of the study team as the findings were being written up, to build a degree of consistency in overall interpretation.

## Results

In total, 400 women enrolled over a 4-month accrual period were followed-up for 12 months from May 2006 to September 2007, with 92% (367/400) retained at 12 months.

### Socio-demographic characteristics

Mean age of participants at enrolment was 25.1 years (SD = 5.2; Table 1). The majority of women (70.1%) had not attended secondary school. The average age of first sex was 17 years, and women had been sex workers for a median 4 years. Half the participants (49.8%) reported only part-time sex work. Weekly income from sex work was ≤500 KSHs ($5.65 USD) for 23.9% of women.

**Table 1:** Socio-demographic characteristics of 400 female sex workers in a prospective cohort study in Mombasa, Kenya, stratified by use of modern contraceptives at baseline.

Variable	All women (n = 400), % (n/N)*	Currently using modern contraceptives (n = 228), % (n/N)*	Currently NOT using modern contraceptives (n = 171), % (n/N)*	P
Socio-demographic and sex work characteristics				
**Age** mean years (sd), n = 398	25.1 (5.2), range = 16–35	25.9 (5.1)	24.1 (5.2)	<0.001^§^
**Religion**				0.86
Catholic	25.5 (102/400)	24.6 (56/228)	26.9 (46/171)	
Protestant or other	39.8 (159/400)	40.4 (92/228)	38.6 (66/171)	
Muslim	34.8 (139/400)	35.1 (80/228)	34.5 (59/171)	
**Highest education level**				0.061
None or primary incomplete	42.3 (169/400)	38.6 (88/228)	47.4 (81/171)	
Primary school	27.8 (111/400)	26.8 (61/228)	28.7 (49/171)	
Secondary or tertiary level	30.0 (120/400)	34.7 (79/228)	24.0 (41/171)	
**Marital status**				0.17
Single	72.0 (288/400)	68.4 (156/228)	76.6 (131/171)	
Married or cohabiting	2.8 (11/400)	3.5 (8/228)	1.8 (3/171)	
Separated, divorced or widowed	25.3 (101/400)	28.1 (64/228)	21.6 (37/171)	
**Age at first sex**, median years (IQR)	17 (15.5–19)	17 (16–18)	17 (15–19)	0.36^¥^
**Sex work duration**, median years (IQR)	4 (2–7)	4 (2–7)	3 (2–6)	0.56^¥^
**Part-time sex work**	49.8 (199/400)	48.7 (111/228)	51.5 (88/171)	0.58
**Weekly income from sex work**				0.96
≤500 KShs	23.9 (95/397)	23.0 (52/226)	25.3 (43/170)	
501–1000	30.2 (120/397)	31.0 (70/226)	29.4 (50/170)	
1001–2000	26.2 (104/397)	26.6 (60/226)	25.9 (44/170)	
>2000	19.7 (78/397)	19.5 (44/226)	19.4 (33/170)	
**Currently has emotional partner**	71.6 (285/398)	66.1 (150/227)	78.8 (134/170)	0.005
**Level of control in relationship with emotional partner**				0.46
High (score 37–48)	16.0 (45/282)	18.1 (27/149)	13.6 (18/132)	
Medium (score 25–36)	46.8 (132/282)	47.7 (71/149)	46.2 (61/132)	
Low (score 12–24)	37.2 (105/282)	34.2 (51/149)	40.2 (53/132)	
Reproductive characteristics				
**Ever used contraception** (multiple response)		–	–	–
Never	4.3 (17/399)			
Long-acting reversible (IUD or implant)	6.8 (27/399)			
Injectable	54.9 (219/399)			
Oral contraception	35.6 (142/399)			
Condoms	93.2 (372/399)			
Other method	14.8 (59/399)			
**Current contraception use**		–	–	–
None or traditional method only	4.3 (17/399)			
Modern method^¶^	57.1 (228/399)			
Long-acting reversible	5.3 (12/228)			
Consistent condom use as only method	19.7 (45/228)			
Inconsistent condom use (+/- traditional method) only	38.6 (154/399)			
**Current dual method use**	10.0 (40/399)	–	–	–
**Number of live children**				<0.001
0	19.8 (79/400)	11.8 (27/228)	29.8 (51/171)	
1	38.0 (152/400)	41.2 (94/228)	33.9 (58/171)	
2–3	34.0 (136/400)	39.0 (89/228)	27.5 (47/171)	
≥4	8.3 (33/400)	7.9 (18/228)	8.8 (15/171)	
**Lifetime abortion** one or more	21.0 (84/400)	21.5 (49/228)	20.5 (35/171)	0.80
Other characteristics				
**Alcohol drinking pattern**				0.058
Alcohol abstinence	36.3 (145/400)	34.7 (79/228)	38.6 (66/171)	
Low-risk drinking	37.0 (148/400)	39.5 (90/228)	33.3 (57/171)	
Hazardous drinking	17.3 (69/400)	19.3 (44/228)	14.6 (25/171)	
Harmful drinking or dependence	9.5 (38/400)	6.6 (15/228)	13.5 (23/171)	

^*^unless otherwise indicated.

^¶^ Current modern contraceptive methods consist of oral contraceptives, implant, injectables, intra-uterine devices (IUD), male and female condoms (self-reported consistent use with all partners over the past 3 months), sterilization, Lactational Amenorrhoea Method, and emergency contraceptives.

^§^Student’s t test.

^¥^Wilcoxon rank-sum test.

Single women made up the majority of the cohort (72%), with only 2.8% married or cohabiting (11/400), and 25.3% (101/400) separated, divorced or widowed. However, 71.6% (285/398) of women reported currently having an emotional partner. Over one third (37%) of those with an emotional partner had low levels of control in their relationship. Most participants (80%) had one or more living children.

### Contraceptive use and attitudes towards use

The majority (96%) of women reported having ever used contraceptives. The most common contraceptive reported was condoms (93%), followed by injectables (55%). Seven percent of women reported having ever used a LARC. At enrolment, just over half (57%; 228/399) were using modern contraception, of which 12 women (5%) reported using a LARC. Only 10% of the women reported current use of dual contraception for prevention of pregnancy and HIV/STIs. A large minority of women were inconsistently using condoms, either alone (36%) or in combination with traditional methods (9 women; 2%).

In the qualitative component of the study, participants generally expressed positive views towards family planning and described the decision to use it as “wise”, an important strategy to plan the size of one’s family and provide for one’s dependents, as well as a way to avoid community ridicule for bearing (more) children as sex workers. Participants often noted that they were the sole providers for their children, and that increasing the burden by having more children would be imprudent, as this exchange in a focus group discussion illustrates:

Dalila: “…With FP [Family Planning] you are able to take care of the children you already have. Then you are able to give yourself time or a chance to plan your life.”

Fathiya: “…It is good to plan because maybe you only have enough resources to take care of two children. Then if a pregnancy occurs, you know like us FSWs having a small child really affects our work since one can’t work then. You have to take care of the small child and hence the others suffer since you have no one to help you.”

A number of participants cited side-effects as a reason for not using contraception. Side-effects described in the focus group discussions included perceptions of "spoiling the uterus", becoming infertile, having backache, "causing itching in the private parts", expansion of growths, rapid pulse, the vagina becoming very wet or smelling badly, and having no libido, while one in-depth interview participant noted that babies that are conceived after using contraception would be born with deformities. Participants in the in-depth interviews and focus group discussions expressed fears about how these side-effects would impact on their work, and were particularly fearful of frigidity, as this dialogue in a focus group discussion illustrates:

Mercy: “…from what she has said, if you plan your family and you are a FSW, and you use either the pills, if you sleep with a client he will tell you: “you are ‘cold’ (frigid).” So on my side, I think it is good that one doesn’t use FP methods because business will dwindle.”

Awiti provided another justification: “…For example, since I am a FSW, then I get injected, then I have sex with a client, he won’t enjoy it like he would with a FSW who doesn’t use FP methods. Hence men will run away from you and you won’t have good business. You will be watery and frigid.”

One participant implicitly evoked how sex workers have a competitive advantage over wives who have lost their libido apparently due to contraception, and thus that sex workers would not want to be placed in the same position:

*Esther*: *“…Some say when a lady uses injection family planning* anakuwa baridi *(laughter)… One of my clients is married and I asked him the reason for moving around with women and he told me that his wife is ‘baridi’*.*”*

Interviewer: “Baridi to mean?”

Esther: “To mean that anytime they have sex, the wife is never turned on even if he touches her so he gets disappointed and he has been persuading her not to use it but she can't because she doesn’t want children.”

It is clear that not all participants shared the belief in the range of side-effects associated with contraception as Fathiya remarks in her focus group discussion:

“…Others do not use [contraception] because of hearsay. This is a group [FSWs] that has never used FP and they hear stories about the side-effects the FP methods have caused on them. Obviously they would fear to fall in the same trap and that is why they opt not to use them.”

### Predictors of current use of modern contraceptive

FSWs reporting current use of modern contraceptives were on average older (25.9 years compared with 24.1 years, *P*<0.001) and more likely to have children (*P*<0.001), compared to those not currently using modern contraception (Table 1). About two thirds of the 120 women who had secondary or tertiary education used contraception (n = 79), compared to just over half of the 169 women who did not complete primary school (n = 88; *P* = 0.020). Compared to women who were using a modern contraceptive, those not doing so were more likely to report harmful or dependent alcohol use (14% vs 7%; p = 0.058), and to have an emotional partner (79% vs 66%; *P* = 0.005).

Qualitative inquiries revealed that control of men over sex workers’ lives and over social norms in the deeply patriarchal society of Mombasa’s urban slums cannot be overstated. Participants often noted how they could not use condoms with their emotional partners. Mukondi in her in-depth interview notes the following:

Mukondi: *“…Because we had reconciled with the father of my son and we were together*, *so I could not tell him to use a condom*.*”*

Interview: “What fear did you have from telling him to use a condom?”

Mukondi: *“…He would have perceived me wrong*.*”*

One participant’s partner perceived a request for condoms as an attack on his masculinity and pride:

*Nafula*: *“…You find a man and tell him to wear a condom*. *Aaah*, *he will tell you; ‘I can't buy a sweet and eat it with its wrapper*! *Don’t take me like a small kid*. Usinifanye mimi mshamaba’ *(don’t make me look uncivilized)*.*”*

Another participant employed a variety of strategies to hide her contraception, and had to negotiate the ever-present risk of violence from her partner:

Shani: “It reached a time when he… when he sees them since he wanted me to bear him a child… I was also monitoring his behaviour that was not good…so he used to throw them [contraceptives] away whenever he found them in my handbag until I used to hide them, even if it was at my neighbour’s house so that when I came in the evening I would take and sleep.

Interviewer: “What were his behaviours?”

Shani: “Just for something minor, he would beat you… beat you…it’s like his wishes were that one of fighting…”

Zawadi also noted the risks of disclosing family planning use in her in-depth interview:

Interviewer: “…Okay. If a partner is unaware of FP use what could happen if he somehow got to know about it?”

Zawadi: “He will withdraw his support because he will see that you are using FP, so there’s no importance of him being with you.

Aluna noted how her boyfriend made her stop contraception as a precondition to continuing their relationship, and she noted her prospects of marriage served as a counter to contraception use:

Aluna: “I am not using any FP method because I got a man who is serious with marriage until he came to our home, knew my mother, so in the state of being together, he wanted me to get pregnant, but we have normally have sex once in a month because he travels a lot.”

### Unintended pregnancies

At enrolment, some 82% (321/400) of sex workers reported to have one (38%) or more (42%) children (Table 1). During the 12-month follow up, a total of 103 pregnancies occurred in the 400 FSWs enrolled in the study. At enrolment, only 2 reported an intention to become pregnant with their emotional (n = 1) or casual partner (n = 1). During the 12-month follow-up, a total of 14 women reported a pregnancy intention at one or more time points. All but one of the women who intended pregnancy wanted their emotional partner to father the child. Of the 14 women wanting pregnancy, 9 became pregnant (data not shown). Therefore, *unintended* pregnancies occurred among a quarter of participants (94/386).

Earning a slightly higher weekly income from sex work of 1001–2000 KShs [US$10–19] was associated with a higher risk of unintended pregnancy (AOR = 2.4, 95%CI = 1.2–4.8; *P* = 0.017) than those earning under 500 KShs (Table 2). Having an emotional partner (AOR = 1.9, 95%CI = 1.0–3.5; *P* = 0.045) and being of a younger age (AOR = 3.0, 95%CI = 1.4–6.5; *P* = 0.008 comparing ≤24 years versus 30 years and older) were also significant independent predictors of unintended pregnancy (Table 2).

**Table 2:** Predictors of unintended pregnancy during 12-months follow-up among 386 Kenyan female sex workers who did not report an intention to become pregnant.

Variable	Proportion of FSWs with unintended pregnancy row, %(n/N)	Crude odds ratio (95%CI)	Adjusted odds ratio (95%CI)	*P*
Socio-demographic and sex work characteristics				
**Age group, years**				**0.008**
≤24 years	31.4 (59/188)	3.5 (1.6–7.3)	3.0 (1.4–6.5)	
25–29 years	22.7 (25/110)	2.2 (1.0–5.0)	2.5 (1.1–5.8)	
30 years and older	11.6 (10/86)	1.0	1.0	
**Religion**				
Catholic	16.7 (16/96)	1.0	1.0	
Protestant or other	26.9 (42/156)	1.8 (1.0–3.5)	1.9 (0.9–3.7)	0.071
Muslim	26.9 (36/134)	1.8 (0.9–3.6)	1.8 (0.9–3.7)	0.087
**Highest education level**			–	0.58
None or primary incomplete	26.1 (43/165)	1.0		
Primary school	25.5 (27/106)	1.0 (0.6–1.7)		
Secondary or tertiary level	20.9 (24/115)	0.7 (0.4–1.3)		
**Sex work duration**			–	0.42
≤2 years duration	27.4 (34/124)	1.0		
3 or 4 years duration	26.3 (26/99)	0.9 (0.5–1.7)		
5 or more years duration	21.1 (34/161)	0.7 (0.4–1.2)		
**Part-time sex work**				0.37
No (full-time)	27.5 (53/193)	1.0	1.0	
Yes	21.2 (41/193)	0.7 (0.4–1.1)	0.8 (0.5–1.3)	
**Weekly income from sex work**				
≤500 KShs	19.2 (18/94)	1.0	1.0	
501–1000	18.6 (22/118)	1.0 (0.5–1.9)	1.0 (0.5–2.0)	0.96
1001–2000	35.4 (35/99)	2.3 (1.2–4.5)	2.4 (1.2–4.8)	**0.017**
>2000	25.0 (18/72)	1.4 (0.7–3.0)	1.3 (0.6–3.0)	0.49
**Currently has emotional partner**				**0.045**
No	16.5 (18/109)	1.0	1.0	
Yes	27.5 (76/276)	1.9 (1.1–3.4)	1.9 (1.0–3.5)	
**Level of control in relationship with emotional partner**			–	0.80
High (score 37–48)	28.9 (13/45)	1.0		
Medium (score 25–36)	25.2 (32/127)	0.8 (0.4–1.8)		
Low (score 12–24)	28.7 (29/101)	1.0 (0.5–2.2)		
Reproductive characteristics				
**Contraception use at baseline¥**				
Modern contraceptive, excl only condom	15.6 (28/180)	1.0	1.0	
Male or female condoms alone	31.7 (60/189)	2.5 (1.5–4.2)	2.7 (1.6–4.7)	**<0.001**
None or traditional method only	31.3 (5/16)	2.5 (0.8–7.7)	2.9 (0.8–9.9)	0.096
**Number of live children**				0.92
0	32.4 (24/74)	1.0	1.0	
≥1	22.4 (70/312)	0.6 (0.3–1.1)	1.0 (0.5–2.0)	
**Lifetime abortion**			–	0.94
No	24.4 (75/307)	1.0		
Yes	24.1 (19/79)	1.0 (0.5–1.7)		
Other characteristics				
**Alcohol drinking pattern**			–	0.34
Alcohol abstinence	23.9 (34/142)	1.0		
Low-risk drinking	23.6 (34/144)	1.0 (0.6–1.7)		
Hazardous drinking	31.3 (21/67)	1.5 (0.8–2.8)		
Harmful drinking or dependence	15.2 (5/33)	0.6 (0.2–1.6)		

Values for the predictor variables are from participants at baseline.

**¥**Current modern contraceptive methods consist of oral contraceptives, implant, injectables, intra-uterine devices (IUD), male and female condoms (self-reported consistent use with all partners over the past 3 months), sterilization, Lactational Amenorrhoea Method, and emergency contraceptives.

In the qualitative discussions, participants constructed marriage as a source of security and financial assurance–indeed, if a sex worker was married, then an unintended pregnancy was not viewed as particularly problematic as the husband was assumed to provide. Barongo in her focus group discussion mentioned:

“FSWs are of many types. There are FSWs like me who have husbands. Therefore giving birth is not an issue, he will take care of the baby, isn’t it? So we are different, like me, I will give birth for my husband because he wants children.”

Moreover, use of only male or female condoms (AOR = 2.7, 95%CI = 1.6–4.7; *P*<0.001) or the use of traditional or no methods (AOR = 2.9, 95%CI = 0.8–9.9; *P* = 0.096) at enrolment were also predictors of unintended pregnancy during follow-up. Due to the nature of these coitus- and user-dependent contraceptives, ‘forgetfulness’ was a frequently cited reason for failure. Shani in her in-depth interview said:

“*I was using [contraception]*, *but sometimes you can forget just like the way I am explaining*. *You could sometimes forget and realize you have involved yourself in a sexual act*”

Aluna, another sex worker noted:

“But as for me, I can say it was foolishness on my side because I was having a method to avoid pregnancy but I never gave it a follow-up you see. It was available, but I had never given it a follow-up. That is why it [unintended pregnancy] happened like so I can say to me I was a fool…Pure foolishness.”

Participants in the in-depth interviews and focus group discussions gave the following examples of incorrect use or method failure that could have led to unintended pregnancies: bursting of condoms, clients who removed condoms just before sex, contraceptive pills taken only on the days that sex takes place, and the belief that the injection's protective efficacy lasts long after the date that she has to return to the clinic. Another frequent explanation was that the pregnancy was “bad luck”–thus suggesting that it was something beyond the participant’s control and subject to chance or (mis)fortune.

### Consequences of unintended pregnancy

Unintended pregnancy posed a range of risks to participants' work as this focus group discussion exchange indicates:

Alice: “To get pregnant is to ask for trouble”

Naomi: “Problems, you won’t be able to take care of the children, your means of livelihood will diminish, since by the time you give birth, all clients will run away from you, even if you have just one client, he might become malicious and stop supporting you since he knows you are an FSW and had many partners, he’s not sure the pregnancy is his. Others will laugh at you for being pregnant and others will ask you why you don’t take care of the kids you have instead of getting others…”

Zawadi also noted some negative direct effects of pregnancy which impacted on her work:

Zawadi: “…and also when pregnant, I always felt moody and like not having sex so I had to stop my sex work. Also when I had sex I bled a lot.”

Interviewer: “Mmmmm…you mean because of morning sickness?

Zawadi: “Yes, I always felt like throwing up always, so I could not serve my clients and that meant I didn’t get money.”

In a context where sex work is highly stigmatised, a visibly pregnant sex worker would attract additional social scorn and judgement. Many participants noted that pregnancy precluded generating an income. Grace in her in-depth interview stated:

Grace: “I will just have to sit idle as I will not be doing my job again”

Several participants noted that clients were offended by pregnancy "because no client will come to you when he sees your tummy bulging" (Shani). In contrast, one participant noted that pregnancy increased her attractiveness to clients: “When I was pregnant I used to get them a lot because they claimed I was very hot” (Janet).

Emotional partners were reported to not be very reliable and liable to abandon women when they found out that they were pregnant. This placed women in a particularly precarious position as use of contraception may lead to abandonment, while paradoxically, becoming pregnant may elicit a similar response. In such a context, it may be pragmatic for FSWs to not use contraception, or not attach significant value to using it consistently, and to find means to abort the pregnancy when they ascertain that their partners do not support the pregnancy sufficiently:

Faith: “[my partner] was very happy because he wanted a baby.”

Interviewer: “So he was happy?”

Faith: “Very much and hoped that it could be twins!”

Interviewer: “Was he supportive of you financially or emotionally?

Faith: “At first he was very happy but later on, it became a burden to him as in he thought twice and thought it could become a burden, especially when I started asking him for financial assistance.”

Interviewer: “Mmmm…so what kind of support did he offer you?

*Faith*: *“Okay*, *he supported me in buying food*, *feeding the other children and also for me*. *But still not sufficiently*. *About finances I had to cater for my other needs*, *so in the early months I stayed in ‘*ubangaizaji’ *(sex work) and because I saw I wouldn’t make it*, *I decided to abort*.*”*

### Abortion

In the prospective cohort, reported lifetime abortion rate was 21% (84/400).

Many in-depth interview and focus group discussion participants expressed strongly negative views on abortion including interpretations of abortion as ‘a sin’, ‘rejecting God’s will for a child’, and ‘fearing disapproval or rejection’ by family and emotional partners. Some participants noted the dangers of abortion and in particular, the risk of death and the subsequent implications for remaining dependents:

*Janet*: *“Let's say I wanted to abort*, *but my mother disapproved of it and told me that many people die because of abortions and you could also destroy your “*kizazi” *[lineage] that's when I decided to just persevere [with the pregnancy]*.*”*

Others, like Aluna, decided that abortion was worth the risk in the longer-term:

*Aluna*: *“I think it is something…it is something I had never done before and I pray not to do it again because it disgusts me a lot (*ilinisinya sana), *because I wouldn't have liked to do such a thing*, *but I think I didn't have the ability to keep that pregnancy…and at that time a lot of thoughts about life*, *you see*. *(*in-depth interview *20)*

Several participants saw abortions through despite their partners’ resistance and the risk of losing their support.

Interviewer: “…and what role did your boyfriend play in facilitating the abortion or against?”

Shani:” …He was very much against me having the abortion, I told you he is poor, but I love him. He told me if I aborted I would die, but for me I told myself, I had to try my best because a baby was an added burden, so there was no way I would keep the pregnancy.”

Esther kept the abortion a secret in the fear that her partner would retaliate:

Interviewer: “Okay…and do you know which partner made you pregnant?”

Esther: “Yes.”

Interviewer: “Did you tell him?”

Esther:”No, because he would have brought chaos and violence thinking that I had aborted his baby, so I did not see any reason of telling him, but he sent me money for hospital bills.”

Participants were asked to describe methods of abortion that they had heard of. Some vividly described injections by biomedical doctors or “sucking machines”. Other strategies included inserting sharp objects (such as pipes, needles, or an umbrella spike) into a pregnant woman’s womb (usually by the woman herself), ingesting malaria drugs or an overdose of contraceptive pills, or taking”local means” or substances prepared by”local people” that included bitter herbs such as *subili* and *neem*, as Maggie described:

Interviewer: “Okay, and how do women not wanting to keep pregnancies procure abortion?”

Maggie: “Some go to the hospital, also others do it at home where they use ‘subili’, ‘muarubaini’ (neem), and others use Omo [brand of washing powder].”

Interviewer: “Mmmm…any other method?.

Maggie: “Others buy medicines from chemists and use an overdose. Others use long sharp objects like sweater needles, they insert which then causes the baby to come out.”

Interviewer: “…and what about cost?

*Maggie: “At the hospital*, *definitely you have to pay large amounts of money depending on the age of the pregnancy*. *But at home there are no costs unless you bleed to death or other complications*.*”*

## Discussion

Unmet need for family planning and the incidence of unintended pregnancy among FSWs has been reported to be high in various settings[7, 9, 10, 33–35]. This was reflected in our study, where about a quarter of FSWs had an unintended pregnancy during the year of observation. More generally, it has been shown that reducing women’s unmet need for contraception could prevent 52 million unintended pregnancies, result in 24 million fewer (often unsafe) abortions, and ultimately prevent 70,000 maternal deaths and 500,000 newborn deaths globally every year[36].

In our cohort of women, some 43% were not using any modern contraceptive method at baseline, remarkably high for a population that predominantly reports not wanting to become pregnant, and higher than other research in this setting[11]. Although contraceptive uptake among FSWs is constrained by the limited availability of sensitive and non-judgmental FSW-specific contraceptive services[19, 37], previous research shows that contraceptive use is also significantly affected by contraception knowledge and perceptions among FSWs in Kenya[11]. Similarly, a study among FSWs in Madagascar showed that low knowledge of contraceptive effectiveness and low self-efficacy were the main predictors for not using contraception[34]. Encouragingly, in a recent study among FSWs in Nairobi Kenya, levels of contraception were higher than reported in our study, although women who reported inconsistent condom use as their only method were included.[38]

In previous mixed-methods formative research, involving 300 FSWs from Mombasa, FSWs’ reported a preference for contraception methods that do not require daily use, such as injectable contraception, and long-acting reversible methods, such as implants or IUDs[11]. In the study reported here, many FSWs seemed to have positive views towards contraception. Despite the desirable attributes of LARC methods (such as not being user- or coitus-dependent) and their reported acceptability among FSWs, only 12 women were using a LARC at the time of enrolment, and only 27 had ever used one. Many are unfamiliar with these methods and their advantages, and they are surrounded by misconceptions about side-effects and safety (e.g. fear of losing libido or of displacing IUD through rough sex), as well as a lack of culturally-sensitive marketing to allay concerns[11, 39, 40]. In this study, women reported similar concerns about side effects, with fear of infertility, congenital anomalies and loss of libido.

Condom use is essential to prevent HIV and other STIs. However condoms are not as effective as other modern methods of contraception at preventing pregnancy, due to often inconsistent or incorrect use. In our study, the incidence of pregnancy among women using condoms *only* (32%) was higher than that in contraceptive efficacy guidelines based on ‘typical use’ (18–21%)[41]. Inconsistent use among FSWs has similarly been demonstrated by previous research with FSWs in Kenya[8, 11, 15, 16, 42] and other countries[7, 34, 43, 44]. FSWs face multiple barriers to consistent condom use, including limited negotiation power with paying and non-paying partners[6, 8, 45], which may be exacerbated by the need to support dependents[43]. Our research showed these difficulties are especially heightened with non-paying emotional partners, with up to 90% of women reporting recent inconsistent condom use with their emotional partners[8, 16, 27, 45], who account for most unintended pregnancies[11, 17, 27]. Reluctance to use condoms because of the implications for trust within a relationship is common in the literature from sub-Saharan Africa[27, 46–48], and the psychological need for FSWs to differentiate between trusting and transactional relationships may also impact on FSWs willingness to use condoms consistently with their emotional partners [49].

Although consistent condom use for HIV prevention needs to be prioritised, dual protection with an additional modern method is critical to prevent unwanted pregnancies and their consequences. This is particularly recommended for high-risk populations, for whom dual protection is the most effective method of preventing both HIV and unintended pregnancy. Our previous research showed that FSWs do recognise the benefit of dual protection for disease and pregnancy prevention[11].

Promoting contraception knowledge, improving attitudes and increasing partner discussion through health communication interventions raises contraception use[40, 50]. Health promotion theories indicate that improving knowledge is a necessary precursor to behaviour change, but addressing health beliefs, norms, and self-efficacy is also essential for healthy behaviour[51, 52]. A trial among HIV-positive couples in Zambia showed the impact of contraceptive promotion on increasing contraceptive uptake and decreasing pregnancy incidence through an intervention of two demonstration videos.[40] The authors concluded that repetition and sustained messaging beyond that trialled would likely further increase comfort with unfamiliar LARCs. Providing contraception information and addressing barriers to contraception uptake through mobile phones could offer a new way to reach and engage FSWs, encourage method use, and establish repeated and low-cost exposure, as has been the case among other target populations such as young people[53].

In our study, nearly three-quarters of women reported having an emotional partner. The attitudes and behaviour of emotional partners heavily influenced women’s contraceptive decision-making and use. Interestingly, the level of control women had over their relationship did not influence pregnancy incidence. In a number of cases, women did not disclose contraceptive use to their partner out of fear of violence and abandonment, similar to other reports in this setting [46]. In some cases, contraceptive use was discussed, but often unwanted by emotional partners due to perceptions of mistrust, particularly if the relationship could evolve into a marriage. However, men were not always willing to support a child should unintended pregnancy occur.

Several factors were identified as being independent predictors of unintended pregnancy, including having an emotional partner, younger age, and using traditional or no contraceptive methods or only condoms. Women in the youngest age group were three fold more likely to have an unintended pregnancy than women in the oldest group. Increased risk-taking among younger women, which has been described elsewhere[18], is of particular concern and could reflect poor understanding and knowledge of contraceptive methods or increased reliance on trust in their sexual partners. Also, risk of unintended pregnancy was associated with higher level of income generated from sex work. Higher income possibly signals more partners, higher payments for condom-free or anal sex, or perhaps relatively wealthier women’s increased capacity to cater for children.

Interestingly, the occurrence of an unintended pregnancy was perceived by some as “bad luck” or “beyond their control”. Many participants seemed to ascribe pregnancy to external loci of control, rather than to the protective potential of contraception or their own agency in this. This signifies more complex issues and power relations that have to be taken into account–most notably the influence of men over contraceptive choices and child-bearing, women’s notions of self-efficacy, and social and cultural understandings of contraception–all within the highly stigmatised and precarious context of sex work.

For a minority of participants, being pregnant while in an emotional relationship allowed women to receive the financial support they needed during pregnancy. Some saw this as an opportunity to evolve their relationship into marriage. However, support did not always materialise, and some participants were abandoned by their emotional partners during pregnancy. In most situations, unintended pregnancies were also unwanted as pregnancy negatively impacted on the ability of sex workers to do their jobs due to the additional stigma of being a pregnant sex worker, or being perceived as less sexually attractive.

Concerning findings regarding abortion in our study highlight the urgency of addressing the high risk of pregnancy in this population and the need for more responsive reproductive health services, including provision of safe termination and counselling. Over 20% of the cohort had ever had an abortion, and women felt conflicted about how to address unintended pregnancy in an environment where termination of pregnancy is highly legally restricted and socially stigmatized. Procuring an abortion through the health system was described as expensive, perhaps due to it taking place in private facilities and the legal risk that practitioners are taking. Participants’ graphic descriptions of self-induced and unregulated terminations, despite their own moral and religious objections and knowledge of the associated dangers, illustrate the extent of the psychological and physical damage that result from unintended pregnancy in this population.

## Conclusion

Uptake of modern contraceptives, particularly dual methods, is low in this population, especially among young women, and results in a high rate of unintended pregnancy. The latter gives rise to financial deprivation, additional social stigma and potential abandonment, as well as the risks associated with self-induced and backstreet abortions. FSWs face considerable barriers to the successful adoption of dual method contraceptive use, including misconceptions and lack of knowledge about methods, and low levels of control in their emotional and commercial relationships. Reducing unintended pregnancy would require addressing these social and structural barriers, including laws that criminalise sex work as well as abortion, and for sex worker-friendly reproductive health services to be more fully incorporated into existing STI and HIV programs for FSWs.
